# *In Vitro* Tumor Models: Advantages, Disadvantages, Variables, and Selecting the Right Platform

**DOI:** 10.3389/fbioe.2016.00012

**Published:** 2016-02-12

**Authors:** Moriah E. Katt, Amanda L. Placone, Andrew D. Wong, Zinnia S. Xu, Peter C. Searson

**Affiliations:** ^1^Institute for Nanobiotechnology (INBT), Johns Hopkins University, Baltimore, MD, USA; ^2^Department of Materials Science and Engineering, Johns Hopkins University, Baltimore, MD, USA; ^3^Department of Biomedical Engineering, Johns Hopkins University, Baltimore, MD, USA; ^4^Sidney Kimmel Comprehensive Cancer Center, Johns Hopkins University, Baltimore, MD, USA

**Keywords:** tumor models, transwell assay, spheroids, metastasis, microvessel models

## Abstract

*In vitro* tumor models have provided important tools for cancer research and serve as low-cost screening platforms for drug therapies; however, cancer recurrence remains largely unchecked due to metastasis, which is the cause of the majority of cancer-related deaths. The need for an improved understanding of the progression and treatment of cancer has pushed for increased accuracy and physiological relevance of *in vitro* tumor models. As a result, *in vitro* tumor models have concurrently increased in complexity and their output parameters further diversified, since these models have progressed beyond simple proliferation, invasion, and cytotoxicity screens and have begun recapitulating critical steps in the metastatic cascade, such as intravasation, extravasation, angiogenesis, matrix remodeling, and tumor cell dormancy. Advances in tumor cell biology, 3D cell culture, tissue engineering, biomaterials, microfabrication, and microfluidics have enabled rapid development of new *in vitro* tumor models that often incorporate multiple cell types, extracellular matrix materials, and spatial and temporal introduction of soluble factors. Other innovations include the incorporation of perfusable microvessels to simulate the tumor vasculature and model intravasation and extravasation. The drive toward precision medicine has increased interest in adapting *in vitro* tumor models for patient-specific therapies, clinical management, and assessment of metastatic potential. Here, we review the wide range of current *in vitro* tumor models and summarize their advantages, disadvantages, and suitability in modeling specific aspects of the metastatic cascade and drug treatment.

## Introduction

*In vitro* and *in vivo* animal models are important tools in cancer research, enabling the identification of carcinogens, the development of cancer therapies, drug screening, and providing insight into the molecular mechanisms of tumor growth and metastasis. In the series of steps that comprise the metastatic process, cancer cells migrate or flow through vastly different microenvironments, including stroma, blood vessel endothelium, the vascular system, and the tissue at a secondary site (Chambers et al., 2002; Fidler, 2003; Steeg, 2006). The ability to successfully negotiate each of these steps is dependent on the interactions between the cancer cell and the local microenvironment (Wirtz et al., 2011). Metastasis is responsible for more than 90% of cancer-related deaths (Weigelt et al., 2005; Mehlen and Puisieux, 2006); however, many details of the steps in the metastatic cascade remain poorly understood (Wirtz et al., 2011).

A wide range of mouse models have been developed of primary and metastatic tumors, including environmentally induced models, human tumor xenografts in immunocompromised mice, and genetically engineered mice (Cekanova and Rathore, 2014; Denayer et al., 2014). While *in vivo* models capture the complexity of the metastatic process in a living system, visualization of the individual steps is challenging and extracting quantitative mechanistic data is usually very difficult. In contrast, *in vitro* models have reduced physiological relevance, capturing only limited aspects of the tumor microenvironment, but allow control of most experimental variables and permit quantitative analysis.

*In vitro* models of solid tumors vary in complexity and range from tumor-derived cell lines to 3D models of the tumor microenvironment (Hulkower and Herber, 2011; Wirtz et al., 2011; Infanger et al., 2013; Vidi et al., 2013). Models have been developed to provide mechanistic insight into tumor growth/proliferation, migration, invasion, matrix remodeling, dormancy, intravasation, extravasation, angiogenesis, and drug delivery. Model variables include cell sources (patient cells, commercially available cell lines, stem cells, stromal cells, immune cells, etc.), biophysical properties (oxygen partial pressure, pH, interstitial flow, etc.), extracellular matrix (ECM) (stiffness, architecture, etc.), and biochemical cues (chemoattractants, angiogenic factors, etc.). The complexity of the model is largely dependent on the objectives. For example, preliminary screening of anticancer drugs can be performed in cell culture. Studies of invasion and motility of tumor cells can be performed with cells embedded in an ECM. Studies of intravasation and extravasation necessitate a microenvironment that incorporates one or more perfusable microvessels.

A key component of any *in vitro* tumor model is a source of cancer cells. Cancer cell lines are easy to grow, allow direct comparison of experimental results, and are widely used to study molecular mechanisms of tumor cell biology (Greshock et al., 2007; Holliday and Speirs, 2011). The molecular profiles of a large number of human cancer cell lines are available in the Cancer Cell Line Encyclopedia (Barretina et al., 2012), and these profiles can be compared to the profiles of a large number of human tumors, compiled as part of the Cancer Genome Atlas Research Network (Holliday and Speirs, 2011; Cancer Genome Atlas Research Network et al., 2013; Domcke et al., 2013). Patient-derived tumorgrafts capture the heterogeneity of cells in a tumor, and in some cases, the tumor histomorphology and global gene expression profile (DeRose et al., 2011); however, engraftment into a mouse or matrix material exerts a selection pressure that changes the clonal composition (Luca et al., 2013; Aparicio et al., 2015). In addition, patient-derived samples provide limited ability for comparison of experimental results. Irrespective of the cell source, models are by definition approximations of a tumor, designed to recapitulate specific aspects of the tumor microenvironment.

Advances in tumor cell biology, 3D cell culture, tissue engineering, biomaterials, microfabrication, and microfluidics have enabled rapid development of *in vitro* tumor models. New models are characterized by increased complexity through the incorporation of multiple cell types (coculture), ECM materials, and spatial and temporal introduction of soluble factors. Here, we review the current state-of-the-art in *in vitro* tumor models. For convenience, models are broadly categorized as transwell-based, spheroid-based, hybrid platforms, and tumor-microvessel models. We summarize the advantages and disadvantages of these models, identify the components of the tumor microenvironment that can be varied, and the phenomena that can be studied (Table 1). This review serves as a guide to selection of *in vitro* platforms best suited to specific applications in tumor biology.

**Table 1:** ***In vitro* tumor models**.

Model	Phenomena
**Transwell-based models**	
Migration	Migration, intravasation, extravasation, drug screening
Invasion	Invasion, intravasation, extravasation, matrix remodeling, drug screening
Transendothelial migration	Intravasation, drug screening
**Spheroid-based models**	
Spheroids in media	Growth/proliferation, drug screening
Spheroids in gels	Growth/proliferation, invasion, matrix remodeling, angiogenesis, drug screening
Coculture	Invasion, angiogenesis, drug screening, immune interactions
**Hybrid models**	
Embedded *ex vivo* tumor sections	Tumor growth, invasion, matrix remodeling, drug screening
3D invasion models	Invasion, matrix remodeling, angiogenesis, dormancy
Avascular microfluidic models	Migration, extravasation
**Tumor-microvessel models**	
Predefined ECM scaffold	Invasion, intravasation, extravasation, angiogenesis, dormancy, drug delivery
Microvessel self-assembly	Invasion, intravasation, extravasation, angiogenesis, dormancy, drug delivery

*Tumor models can be broadly classified as transwell-based, spheroid-based, hybrid platforms, and tumor-microvessel models. Each model has the ability to model different processes in the progression and spread of cancer*.

## Transwell-Based Models

### Introduction

**Figure 1:**
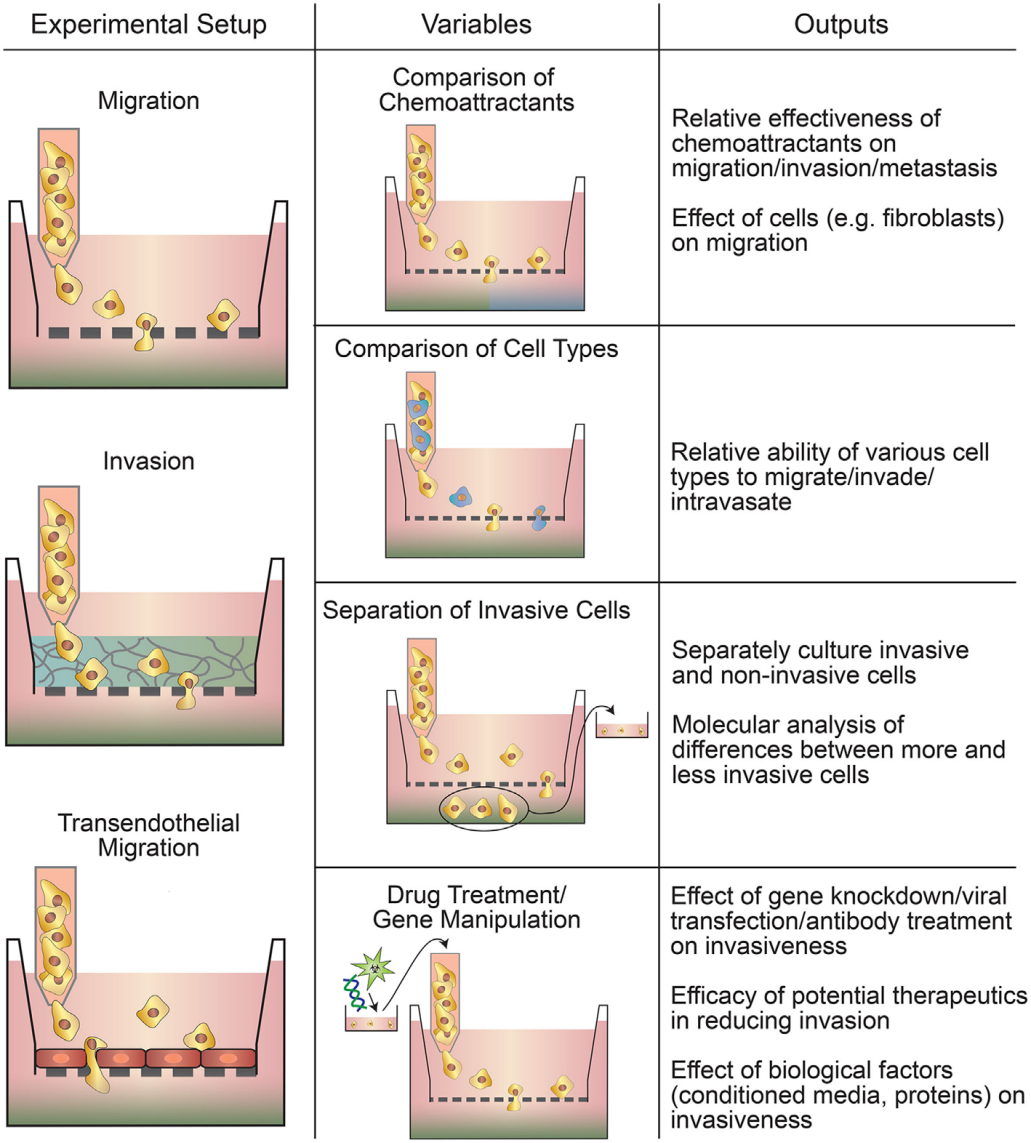
**Types of transwell-based motility assays**. Migration, invasion, and transendothelial migration setups can all be used to assay multiple parameters, such as the relative invasiveness of different cells and the effect of drugs or gene manipulation on motility.

Transwell-based assays are widely used to assess cancer cell migration and invasion (Figure 1) (Hulkower and Herber, 2011; Marshall, 2011; Kramer et al., 2013). Migration is the movement of cells from one location to another and is central to the metastatic cascade (Madsen et al., 2015). Cell migration may be random or directed by gradients in soluble factors, electric field, or matrix stiffness (Rorth, 2009; Madsen et al., 2015). Invasion refers to the migration of cells in a 3D ECM (Friedl and Alexander, 2011; Friedl et al., 2012a). The three commonly used variations of transwell-based assays are (1) migration assays, (2) invasion assays, and (3) transendothelial migration assays (Table 2). Migration, the simplest variation, involves seeding cancer cells directly on a porous membrane, while invasion assays involve seeding cells on a layer of ECM material on top of the porous membrane (Kramer et al., 2013). Transendothelial migration assays involve a confluent layer of endothelial cells on top of the membrane (Smith et al., 1991). Transwell-based assays are usually combined with a chemoattractant gradient, typically media with 10% FBS in the bottom chamber and media with ≤1% FBS in the upper chamber (Marshall, 2011; Kramer et al., 2013). Other common chemoattractants include individual growth factors (Carter and Church, 2012), ECM proteins (Kao et al., 2008), and paracrine signals from other cell types (e.g., fibroblasts) (Underwood et al., 2015).

**Table 2:** **Advantages and disadvantages of transwell-based tumor models**.

Transwell model	Description	Advantages	Disadvantages
Migration	Cancer cells pass through transwell membrane, analogous to leaky endothelium	• Easily implemented, low-cost assay • High throughput • Can be used to compare metastatic potential of cells	• Low physiological relevance • Can only assay single-cell motility • Migration and invasion assays can result in conflicting data
Invasion	Cancer cells pass through a layer of ECM and the transwell membrane		
Transendothelial migration	Cancer cells pass through a monolayer of endothelial cells to assay extravasation		

### Migration Assays

The migration assay involves seeding cancer cells on top of a porous membrane (typically with 8 μm pores) and quantifying the number of cells that can migrate across the membrane toward a chemoattractant (Marshall, 2011). Cells are typically fixed after 24–72 h for imaging. Staining with either a nuclear stain or crystal violet is common for counting the migrated cells (Marshall, 2011). The bottom surface of the membrane is imaged and typically quantified by cells per imaging field. While this is a simplistic assay, the degree of migration through pores toward serum provides a high throughput *in vitro* model of tumor intravasation through leaky vasculature, with the pores in the membrane representing the gaps in the endothelium and serum representing the bloodstream (Pouliot et al., 2000).

### Invasion Assays

Invasion assays add another level of complexity to this model. A layer of ECM is deposited on the porous membrane to model the basement membrane of the vasculature. This layer is typically Matrigel (Marshall, 2011), although collagen (Wang et al., 2010), and laminin (Rath et al., 2013) are also used. ECM layers can range in thickness up to 1 mm (Kramer et al., 2013). While migration and invasion assays probe very similar properties of cells, it is worth noting that some drugs and gene manipulations have a stronger effect on the reduction of invasion than migration. For example, when transfected to overexpress TPFR-2, which inhibits MMP activity, PANC-1 pancreatic cancer cells show no reduction in migration but a 60% reduction in invasion (Tang et al., 2010). In another study, it was found that siRNA knockdown of IMP3 resulted in decreased expression of CD-44, reduced migration by 30% while decreasing invasion by 80% (Pasiliao et al., 2015).

### Transendothelial Migration Assays

Transendothelial migration assays involve plating a confluent monolayer of endothelial cells onto the porous support. This model has the additional complexity of the cell–cell junctions between endothelial cells and the ECM that they produce (Smith et al., 1991). In this configuration, transendothelial assays provide a good model of extravasation, as cancer cells must first pass through the endothelium and then the deposited basement membrane (Rahn et al., 2005). This assay can also be inverted to model intravasation by growing endothelial cells on the bottom side of the transwell membrane to confluence and seeding cancer cells in the top chamber (Pignatelli et al., 2014). These transendothelial assays are most commonly used to study brain capillary endothelium, which have tight cell–cell junctions (Lin et al., 2015), although other endothelial cell types, such as HUVECs (Pignatelli et al., 2014), are used as well.

### Applications of Transwell-Based Assays

Transwell methods are used for drug screening and to study migration, intravasation, extravasation, and matrix remodeling. In most cases, transwell-based assays involve counting the number of cells that are able to translocate across the porous membrane under the experimental conditions. Many tumor cell lines exhibit a threefold to fivefold increase in the number of cells translocating across the membrane compared to non-cancerous cells (Li and Zhu, 1999), while drug and gene therapies typically reduce invasion by 30–80% (Tang et al., 2010; Lin et al., 2015; Yang et al., 2015). Applications of transwell-based assays include (1) studies of the influence of chemoattractants on migration and invasion (Orellana et al., 2015), (2) studies of the influence of other cell types (e.g., macrophages and fibroblasts) on invasion of cancer cells (Pignatelli et al., 2014), (3) studies of the relative rates of invasion, migration, intravasation, and extravasation of different cell types (Li and Zhu, 1999), (4) the isolation of invasive/non-invasive cell types for molecular analysis (Kao et al., 2008), (5) testing the influence of knockdown, transfection, and antibody treatment on invasion and migration (Gan et al., 2015), (6) assessing drug therapies in reducing invasion (Yang et al., 2015), and (7) basic studies of the role of soluble factors on invasion (Carter and Church, 2012). As an example of the application of transwell-based assays, renal cancer cells from patients with bone metastases showed a 20-fold increase in migration toward calcium compared to cells from patients that had not metastasized (Joeckel et al., 2014). This study illustrates how a relatively straightforward *in vitro* assay can be used as a diagnostic tool to assess the ability of a patient’s primary tumor to metastasize to a specific secondary site.

## Spheroids

### Introduction

**Figure 2:**
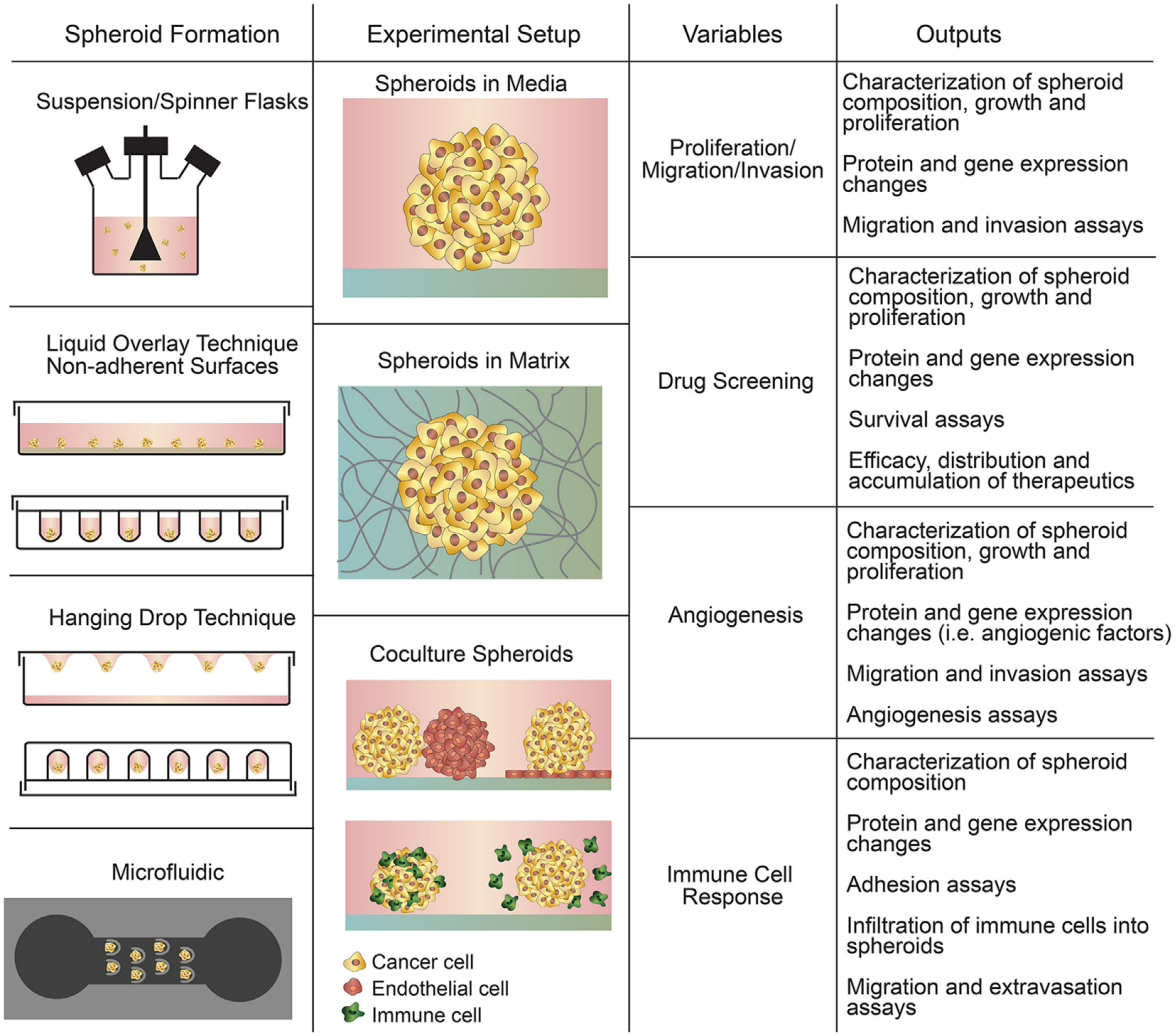
**Summary of spheroid-based assays including spheroid formation techniques, experimental setups, variables to study, and experimental outputs**.

Spheroids are aggregates of cells grown in suspension or embedded in a 3D matrix using 3D culture methods (Figure 2) (Mueller-Klieser, 1987; Gottfried et al., 2006; Hirschhaeuser et al., 2010; LaBarbera et al., 2012; Fennema et al., 2013). Cancer cell spheroids, known as multicellular tumor spheroids (MCTS), represent avascular tumor nodules or micro-metastases (Friedrich et al., 2009). While more expensive and time consuming compared to 2D cell culture, 3D spheroids are widely used for drug screening and studies of tumor growth and proliferation, immune interactions, and for the case of spheroids embedded in a matrix, studies of invasion, matrix remodeling, and angiogenesis (Mueller-Klieser, 1987; Gottfried et al., 2006; Friedrich et al., 2007; Hirschhaeuser et al., 2010; LaBarbera et al., 2012). 3D spheroids recapitulate cell–cell and cell–matrix interactions between tumor cells and the microenvironment (Hirschhaeuser et al., 2010; Mehta et al., 2012; Fennema et al., 2013), as well as transport properties (Mehta et al., 2012). Larger spheroids sustain oxygen and nutrient gradients that often result in the formation of a necrotic core similar to poorly vascularized tumors (Friedrich et al., 2009). Spheroids also demonstrate proliferation gradients and zones reminiscent of tumors (Mueller-Klieser, 1987). As a result of these factors, the protein and gene expression profiles of tumor cells in spheroids are much closer to clinical and *in vivo* gene expression profiles than those in 2D culture (Friedrich et al., 2009; LaBarbera et al., 2012).

An important variable in MCTS is tumor size since it is correlated with cell function, as well as drug penetration and transport. In general, spheroids between 200 and 500 μm are sufficiently large to develop gradients of oxygen, nutrients, and catabolites (Hirschhaeuser et al., 2010). Above a critical size of 400–600 μm, spheroids develop a central secondary necrosis where the innermost cells die of apoptosis or necrosis (Gottfried et al., 2006; Friedrich et al., 2009; Hirschhaeuser et al., 2010). These larger spheroids generally have a viable cell rim that is 100–300 μm thick around the necrotic core (Gottfried et al., 2006). Spheroids as small as 200 μm have been used for drug testing and may be sufficient to recapitulate cell–cell and cell–matrix interactions but are not large enough to recapitulate oxygen gradients with hypoxic regions or proliferation gradients (Friedrich et al., 2009). The time to culture a 400-μm spheroid (seeded at a density of 500 cells per well) is around 4 days but is dependent on cell type (Friedrich et al., 2009). For short-term culture (<48 h), spheroids may not be as densely packed as spheroids that have been cultured for longer and may not recapitulate cell–cell and cell–matrix interactions.

### Spheroid Formation

There are four general methods of spheroid formation (Figure 2), each with their distinct advantages and disadvantages (Table 3): suspension culture, non-adherent surface methods, hanging drop methods, and microfluidic methods (Mehta et al., 2012). Suspension culture methods promote spheroid formation by maintaining cells in suspension through agitation or by increasing the viscosity of the media (e.g., with the addition of carboxymethyl cellulose), thereby enabling spontaneous aggregation (Lin and Chang, 2008; Metzger et al., 2011). Suspension culture has high throughput but does not allow control of size and uniformity (Lin and Chang, 2008; Mehta et al., 2012).

**Table 3:** **Advantages and disadvantages of tumor spheroid models**.

Spheroid-forming method	Description	Advantages	Disadvantages
Cell suspension culture	Spheroids are cultured in suspension to avoid sedimentation and adherence	• Simple • Mass production • Long-term culture	• No individual compartments for spheroids • Cannot control uniformity (size, composition) • High shear force
Non-adherent surfaces	Spheroids are induced to form on planar non-adherent surfaces or microarray wells (MW)	• Simple • Better efficiency (MW) • High throughput (MW) • Uniform spheroid size (MW) • Coculture (MW)	• Low throughput • Long-term culture difficult
Hanging drop technique	Hanging droplets of spheroids from underside of lid	• Control of spheroid size • Uniform spheroid size • Allows coculture with defined cell types	• Low throughput • Long-term culture difficult • Not efficient
Microfluidic devices	Spheroids are generated within microfluidic channels	• Control of spheroid size • Control of spheroid growth parameters • Continuous perfusion • Faster spheroid formation	• Difficulty collecting cells for analysis

Culturing cells on non-adherent surfaces prevents attachment to the substrate and promotes spheroid formation. In the liquid overlay technique (LOT), suspended cells are cultured on a non-adherent surface, such as agar, 1–1.5% agarose, or poly-HEMA (Yuhas et al., 1977; Ivascu and Kubbies, 2006; Metzger et al., 2011). Using non-adherent surfaces is straightforward but does not allow control over spheroid size and uniformity. Growth of spheroids in microarrays greatly increases throughput while allowing control of spheroid size (Hsiao et al., 2012; Mehta et al., 2012; Fennema et al., 2013). Spheroid growth can be directed using round-bottom non-adherent 96-well plates or stamped agarose microwells (Fennema et al., 2013).

The hanging drop method and techniques that employ microfluidic devices are more complex but allow better control of spheroid size and composition (Mehta et al., 2012). In the hanging drop technique, droplets of cells are suspended from the underside of an adherent tissue culture lid. Gravity drives cell aggregation into a cluster at the bottom of the drop, which then grows into a spheroid (Kelm et al., 2003). The hanging drop technique is relatively straightforward and allows for uniform spheroid size but is relatively low throughput, partially due to the necessity of manual media changes.

Microfluidic devices are becoming increasingly common since they allow precise control of spheroid formation (Wu et al., 2008; Mehta et al., 2012; Fu et al., 2014). Continuous perfusion under physiological conditions during spheroid formation allows for faster formation and increased size uniformity (Mehta et al., 2012). Microfluidic platforms also allow the formation, maintenance, and testing of spheroids within a single device (Wu et al., 2008).

### Applications of Tumor Spheroids

Spheroids have been used in four main applications: the study of cell function (e.g., cell proliferation, migration, and invasion) in an avascular tumor microenvironment, the development of new therapies and drug screening, the study of tumor angiogenesis, and the study of tumor–immune cell interactions (Mueller-Klieser, 1987; Gottfried et al., 2006; Lin and Chang, 2008; Hirschhaeuser et al., 2010; Fennema et al., 2013).

#### Cell Function

Early studies of spheroids focused on recapitulating solid tumors and studying growth kinetics (size versus time), composition, and tumor cell biology (e.g., proliferation, differentiation, cell death, protein and gene expression, etc.) (Sutherland et al., 1971; Freyer and Sutherland, 1986; Durand, 1990; Friedrich et al., 2009). Studies comparing gene expression profiles of spheroids and 2D cultures to resected tumors revealed differences in genes associated with cell survival, proliferation, differentiation, and resistance to drug therapy and showed that spheroids more closely resembled *in vivo* tumors (Hirschhaeuser et al., 2010).

The ability of cells to migrate is a hallmark of the epithelial to mesenchymal transition (Gagliano et al., 2005). Cell migration assays have been developed to test therapeutics and their ability to reduce tumor cell migration and inhibit their transition to an invasive, metastatic phenotype (Rao et al., 2005; Vinci et al., 2013). Invasion studies are performed by placing spheroids on coated surfaces (i.e., vitronectin-coated) or embedding in gels (i.e., collagen type I) and measuring their invasiveness, as well as analyzing factors involved in matrix degradation and tumor invasion, such as cathepsin-B and matrix metalloproteinases (MMP) (Tamaki et al., 1997; Lakka et al., 2004; Wolf et al., 2007; Ilina et al., 2011).

#### Drug Screening

Cancer spheroids are widely used to assess tumor response and sensitivity to chemotherapeutics, combination therapies (e.g., chemotherapeutics and small molecule inhibitors), targeted chemotherapy, and drug delivery vehicles (L’Esperance et al., 2008; Perche et al., 2012; Mikhail et al., 2013; Sarisozen et al., 2014). Spheroids are commonly used as a high-throughput tool for negative selection of drug candidates to reduce animal testing (Friedrich et al., 2009) and for positive selection in new drug development (Hirschhaeuser et al., 2010). Drug screening typically involves spheroid formation, incubation with a drug, measurement of spheroid integrity and growth kinetics (growth delay and regrowth), and measurement of cell survival (e.g., acid phosphatase assay and colony formation assay) (Friedrich et al., 2007, 2009; Hirschhaeuser et al., 2010). The colony formation assay is used to measure the ability of a single cell to grow into a colony and is used to assess clonogenic survival (Franken et al., 2006; Hirschhaeuser et al., 2010). Overall, MCTS are more resistant to treatment than cells in 2D culture (Lin and Chang, 2008; Mehta et al., 2012; Fennema et al., 2013) and can recapitulate the drug resistance observed in solid tumors (Friedrich et al., 2009).

#### Angiogenesis

The potential for tumor vascularization is often assessed from the migration of endothelial cells into tumor spheroids or the formation of vascular networks within spheroids (Timmins et al., 2004). Protocols include the culture of MCTS on endothelial cell monolayers, coculture of MCTS spheroids and EC spheroids, and spheroids formed from a mixture of tumor cells and endothelial cells (Jadhav et al., 2004; Timmins et al., 2004; Ghosh et al., 2007; Upreti et al., 2011). Tumor-induced angiogenesis can increase oxygen consumption and increase expression of hypoxia-related and proangiogenic genes (Wartenberg et al., 2001). Other studies have focused on factors that induce or inhibit angiogenesis, such as MMP-9 which plays a key role in endothelial network organization (Jadhav et al., 2004). 3D spheroid coculture models are increasingly used in tissue engineering to modulate angiogenesis (Korff and Augustin, 1998; Korff et al., 2001; Wenger et al., 2004, 2005).

#### Immune Cell Response

The immune system plays an important role in the antitumor response that is primarily driven by natural killer cells, dendritic cells, and macrophages (Hickey and Kubes, 2009; Pardoll, 2012; Gajewski et al., 2013). The tumor-immune response is assessed by culturing MCTS with immune cells and observing the migration and infiltration of immune cells or by forming spheroids from tumor cells and immune cells and observing the interactions and cytotoxic effects of immune cells within tumor spheroids (Gottfried et al., 2006). Tumor cells often secrete factors that induce an immunosuppressive environment. For example, the lactic acid-rich environment surrounding tumors inhibits the cytotoxic activity of T-lymphocytes (CTLs) (Fischer et al., 2007) and reduces the migration of monocytes (Gottfried et al., 2006). Tumor spheroids are also being used to develop therapeutic strategies to stimulate an immune response by promoting infiltration and cytotoxicity of various immune cells (Durek et al., 1999; Hoffmann et al., 2009).

## Hybrid Models

### Introduction

**Figure 3:**
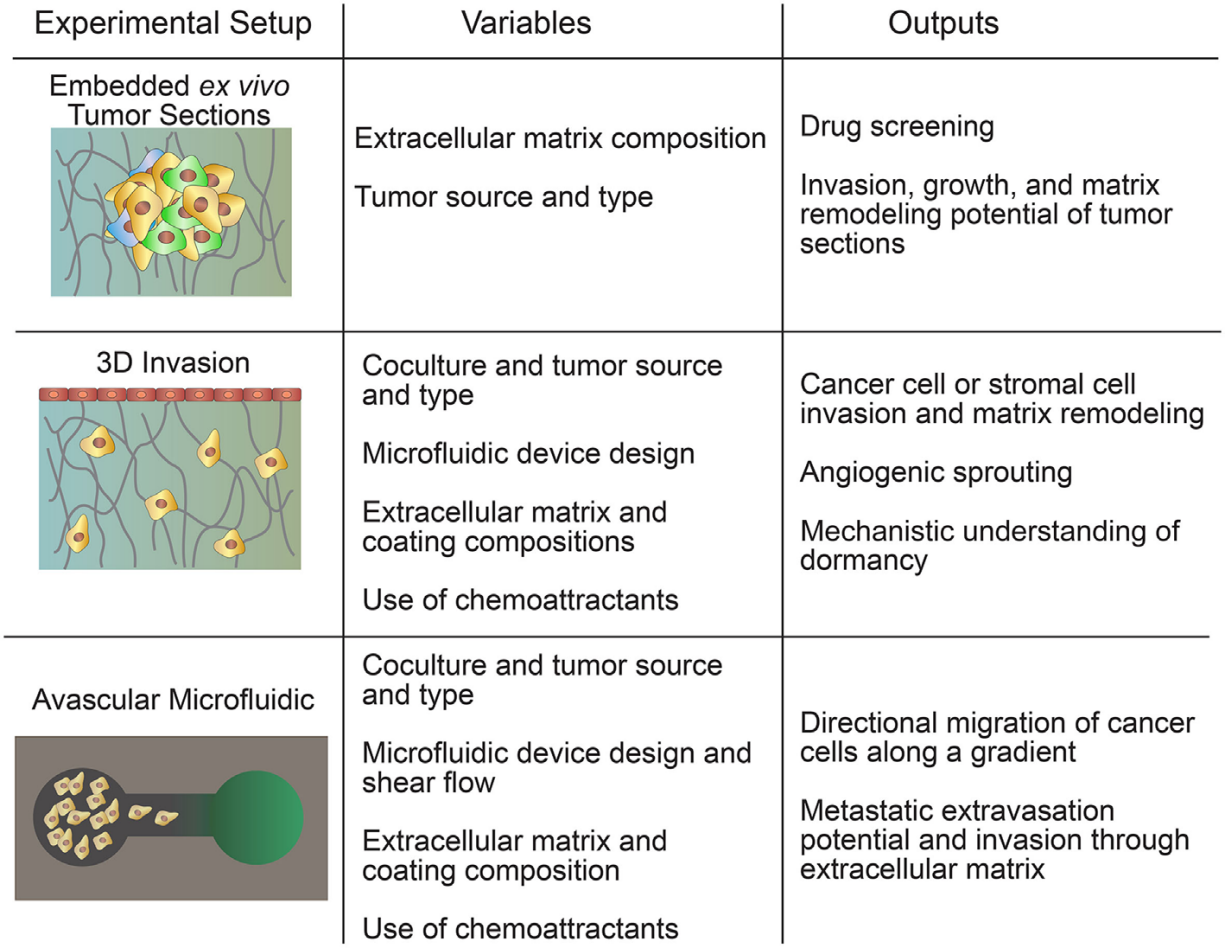
**Hybrid models include embedded *ex vivo* tumor sections, 3D invasion models, and avascular microfluidic models**.

There are several types of *in vitro* tumor models that cannot be classified as spheroid- or transwell-based. These include embedded *ex vivo* tumor sections, 3D invasion models, and avascular microfluidic models (Figure 3). These models combine the complexity of the tumor microenvironment while maintaining the relative simplicity of an *in vitro* model (Table 4). Embedded *ex vivo* tumor sections from patient biopsies can be used to select individualized chemotherapeutic regimens and fundamental studies of tumor growth and invasion (Yabushita et al., 2004). 3D invasion models reduce some of the complexities involved in the embedded biopsy samples by allowing clear visualization of specific cell interactions and interrogation of a wide range of events in the metastatic cascade. Avascular microfluidic models are the simplest in this category but still incorporate a wide range of techniques to interrogate the migration of tumor cells in a variety of geometries.

**Table 4:** **Advantages and disadvantages of hybrid tumor models**.

Model	Description	Advantages	Disadvantages
Embedded *ex vivo* tumor sections	• Primary tumor sections or biopsies embedded in gel	• Maintains tumor heterogeneity • Patient-specific assay • Mimics outgrowth into surrounding tissues	• Lacks flow through vasculature
3D invasion models	• Tumor cells or clusters embedded in a gel	• 3D microenvironment • Allows real-time tracking of cells • Balance of complexity and experimental control	• Lacks vasculature • Lacks tumor complexity
Avascular microfluidic	• Tumor cells grown in a 2D microfluidic device, typically for the study of migration	• Simple migration assay • Easy to isolate effect of variables • Allows real-time tracking of cells	• Lacks vasculature • Typically lacks 3D environment

### Embedded *Ex Vivo* Tumor Sections

The use of tumor biopsies or resected tumors sections embedded in an ECM has been employed to interrogate the tumor microenvironment *in vitro* (Miller et al., 1984, 1986; Dark et al., 1997; Kobayashi et al., 1997; Yamada et al., 1999; Brown et al., 2004; Xu et al., 2013). Embedded biopsies or tumor sections maintain the heterogeneity of tumor cell subpopulations, supporting tissue cells, and the tumor vasculature. While the tumor vasculature is not perfusable in these models, it is a valuable tool for characterization and study as it removes many of the *in vivo* complexities but maintains the cell interactions. This technique is largely used for characterization of tumor morphology, growth, and chemosensitivity (Dark et al., 1997) and has potential as a technique for screening patient-specific therapies (Xu et al., 2013).

Tumor sections are typically embedded in collagen type I as a mimic of the ECM (Nguyen-Ngoc et al., 2012), although it has been shown that the gene expression and phenotypic profiles of the cancer cells are dependent on the matrix material (Kievit et al., 2010; Nguyen-Ngoc et al., 2012). Genes associated with cell adhesion, such as the cadherins, integrins, and lectins, were significantly downregulated in experiments where disseminating cancer cells were isolated from a 3D collagen type I matrix (Nguyen-Ngoc et al., 2012). Embedded tumor sections have been used to characterize the growth and invasion of brain (Tsuchida et al., 1998; Yamada et al., 1999) and mammary tumors (Miller et al., 1984, 1986) and have been used to study drug penetration into the tumor (Netti et al., 2000; Ramanujan et al., 2002; Brown et al., 2004).

The most common use of embedded tissue sections is the culture-drug sensitivity test (CD-DST), where cells from a patient-derived tumor are cultured in collagen droplets and incubated with different anticancer drugs, and the chemosensitivity is assessed from the number of remaining viable cells. This technique has been compared to the outcomes of patients with a wide variety of tumor types (Kobayashi et al., 1997; Hanatani et al., 2000) and is currently in clinical trials as a tool in the patient-specific treatment of cancer (Yabushita et al., 2004).

### 3D Invasion Models

While the embedded spheroid and embedded tumor section models can be used to image global growth, protrusion formation, and detachment and invasion of individual tumor cells, 3D invasion models focus specifically on invasion by seeding individual or clusters of cancer cells in an ECM material, and thus reducing some of the complexities of the tumor microenvironment. Live cell imaging is used to determine cell morphology and track the trajectories of individual cells allowing quantification of cell speed and persistence through the ECM. This approach can be used to study the role of ECM material, matrix stiffness, chemotactic gradients, and hypoxia on cell adhesion, invasion, and matrix remodeling (Liu et al., 2010; Sung et al., 2011; Koch et al., 2012; Kim et al., 2013; Shen et al., 2014; Mosadegh et al., 2015). Coculture variations of 3D invasion models often include a monolayer of fibroblasts, endothelial cells, or cancer cells on a matrix material with cancer cells or fibroblasts embedded in the matrix. These models can be used to study the influence of proangiogenic factors secreted by the tumor cells, as well as chemoattractants secreted by endothelial cells or fibroblasts (Krause et al., 2010; Liu et al., 2010; Shen et al., 2014; Horie et al., 2015).

### Avascular Microfluidic Models

Avascular microfluidic devices are primarily used to assess cancer cell migration along small channels with respect to chemotactic gradients. Many avascular microfluidic devices study migration along small confined channels that are designed to mimic the quasi one-dimensional migration between fibers in the ECM (Fraley et al., 2010; Wirtz et al., 2011; Friedl et al., 2012b; Konstantopoulos et al., 2013). Microfluidic devices allow well-defined gradients of chemoattractants and other molecules across the channels, as well as control of oxygen partial pressure, and other stimuli (Guan et al., 2015). The channels can be coated with different adhesion proteins and/or matrix materials to modulate cell adhesion or can be filled with ECM to simulate confined migration in 3D (Chaw et al., 2007; Hou et al., 2009). As with other *in vitro* tumor models, multiple cell types can also be introduced (Liu et al., 2010; Ma et al., 2010; Gao et al., 2011). Experimental measurements typically involve using live cell microscopy to determine cell speed along the channels as a function of experimental conditions (channel dimensions, the presence of obstacles, coating/ECM materials, solute gradients, etc.) (Chaw et al., 2007; Hou et al., 2009). Studies in microfluidic channels have contributed to the discovery of genes required for cancer cell migration and identification of proteins, such as EGFR or CXCL12, that act as chemoattractants for cancer cells (Saadi et al., 2006; Torisawa et al., 2010).

Microfluidic models have been used to study cancer cell adhesion to endothelial monolayers as a precursor to extravasation. In these models, a monolayer of endothelial cells is formed in a microfluidic channel, and cancer cells are subsequently introduced into the channel over the endothelial monolayer at a fixed flow rate. Using live cell microscopy, the adhesion and rolling of the cancer cells can be studied as a function of shear stress with or without inhibitors or antibodies for adhesion molecules. These studies have shown that E-selectin and CXCL12 are important endothelial receptors for cancer cell adhesion (Tözeren et al., 1995; Khaldoyanidi et al., 2003; Song et al., 2009; Hsu et al., 2011).

## Tumor-Microvessel Models

### Introduction

The tumor vasculature is an important component of the tumor microenvironment providing nutrients essential for growth. The endothelial cells lining blood vessels are known to secrete factors that both promote and suppress tumor growth (Butler et al., 2010; Ghajar et al., 2013). Furthermore, the tumor vasculature plays a critical role in several key events in the metastatic cascade, such as invasion, intravasation, and extravasation. The complex interplay between the tumor vasculature and cancer cells can be studied using *in vitro* tumor models that incorporate microvessels.

**Figure 4:**
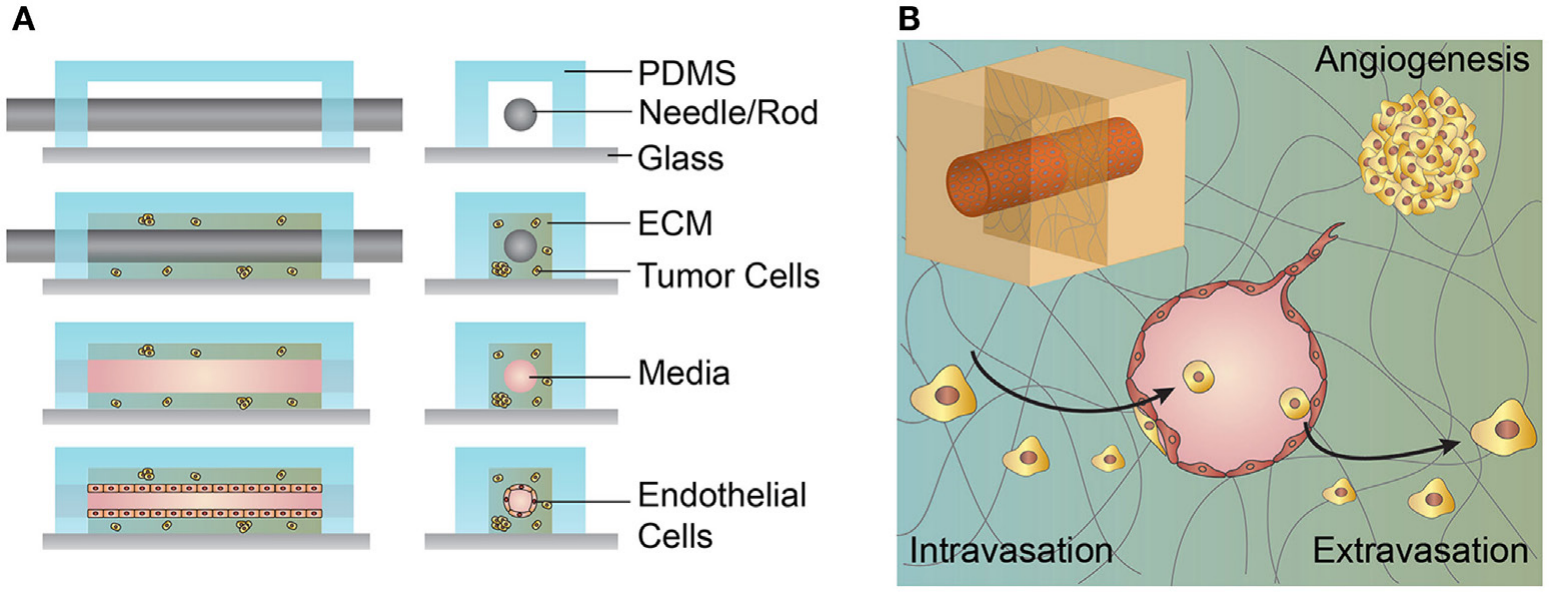
**Schematic illustration of the microvessel fabrication process and interactions between the microvessel and tumor cells in the surrounding extracellular matrix (ECM)**. **(A)** A solution form of ECM, often collagen type I or fibrin, laden with cells is introduced around the cylindrical template within the PDMS housing. After gelation/cross-linking, the template rod is removed. Endothelial cells are introduced and line the interior of the cylindrical channel. **(B)** Upper left inset, cylindrical channel lined with endothelial cells embedded within an ECM. The figure shows a cross-section of a cylindrical vessel interacting with tumor cells in multiple ways. Tumor cells may secrete growth factors and cytokines that promote angiogenesis from a nearby vessel. Tumor cells may invade and intravasate within the local vasculature. Tumor cells within the circulating media may extravasate by adhering to the vessel wall, transmigrating across the endothelium, and invading into the ECM.

Microvessels used to study interactions between tumor cells and the tumor vasculature are generally fabricated by seeding endothelial cells onto predefined ECM scaffolds or self-assembled through matrix remodeling after randomly dispersing endothelial cells within an ECM (Figure 4) (Bogorad et al., 2015). Each approach has advantages and disadvantages depending on the application (Table 5).

**Table 5:** **Advantages and disadvantages of tumor-microvessel models**.

Tumor-microvessel model	Platform	Application	Advantages	Disadvantages
Predefined ECM scaffold	Cylindrical template/scaffold	Invasion and intravasation	• Well-defined vessel endothelium and shear stress	Limited vessel diameter ranges (>50 μm) and simple linear geometries
	Cylindrical template/scaffold	Vessel paracrine signaling with respect to varying shear stresses		
	Cylindrical template/scaffold	Transvascular migration		
	Microfluidic-confined ECM	Intravasation and extravasation		
Microvessel self-assembly	Microfluidic-confined ECM	Extravasation	• Generates vessel sizes from capillaries to small microvessels (5–50 μm) and complex networks	Random vessel network with unpredictable flow
	Microfluidic-confined ECM	Drug toxicity screening		
	Free gel	Tumor cell dormancy		

### Predefined ECM Scaffold

Cylindrical microvessels with diameters as small as 50 μm can be fabricated using subtractive templating methods. These microvessels are generated by seeding endothelial cells on the internal surface of a predefined channel in an ECM, typically collagen type I or fibrin. Cells seeded on these ECM surfaces will self-assemble into a continuous monolayer and can be tested for functional properties, such as vessel permeability, expression of relevant junctional proteins, and appropriate response to vascular mediators and inflammatory cytokines (Chrobak et al., 2006). These 3D cylindrical microvessels exhibit a physiologically relevant geometry, can be maintained under shear stress, and cocultured with a variety of cell types. While it is possible to incorporate smooth muscle cells, pericytes, and lymphatic drainage within these models, the lack of such vessel characteristics is a hallmark of irregularly formed tumor vasculature (Hanahan and Weinberg, 2011; Zheng et al., 2012; Wong et al., 2013). The coculture of endothelial-lined microvessels with tumor cells permits the study of a variety of tumor–endothelial interactions, such as endothelial paracrine signaling, tumor-driven angiogenesis, intravasation, and extravasation (Figure 4) (Buchanan et al., 2014; Wong and Searson, 2014; Wang et al., 2015). The extraction of tumor cells from the surrounding ECM and analysis of their gene expression has shown that tumor cell invasiveness is mediated by the presence of microvessels and vessel shear stress (Buchanan et al., 2014). Live cell imaging of cocultured artificial microvessels with tumor cells in the surrounding ECM has recapitulated interactions thought to occur during cancer metastasis, such as invasion, tumor-driven angiogenesis, intravasation, and extravasation (Wong and Searson, 2014; Wang et al., 2015). Qualitative observations of invasion and intravasation suggest that one mechanism of tumor cell entry into the vasculature is mediated by tumor cell activation and division at the ECM–vessel interface resulting in mechanical disruption of the endothelial monolayer (Wong and Searson, 2014).

**Figure 5:**
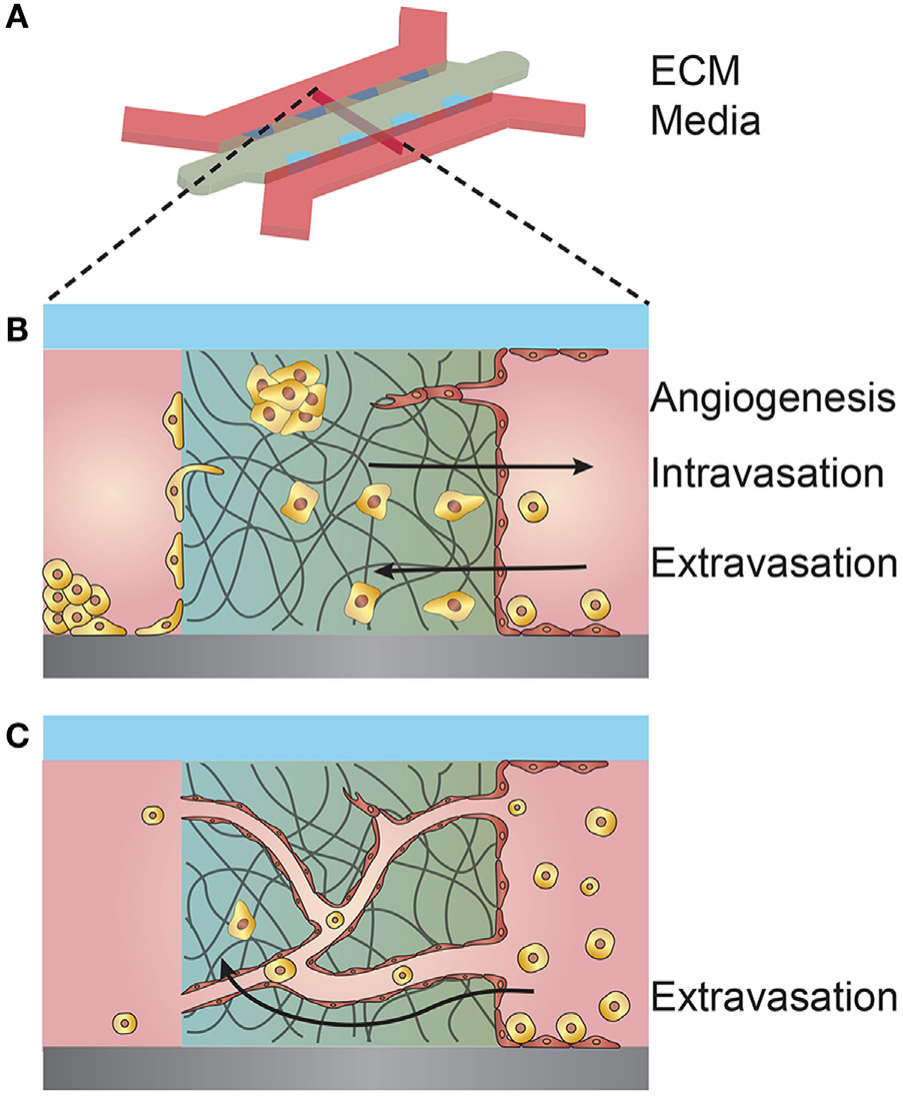
**Schematic illustration of a microfluidic device used for coculturing endothelial and tumor cells**. **(A)** 3D schematic overview of a microfluidic device composed of an extracellular matrix (ECM) confined within PDMS posts and separating two media channels. **(B)** Cross-section of the coculturing device showing multiple interactions between tumor cells and a seeded endothelium. Tumor cells within the ECM may elicit an angiogenic response from the endothelium. Tumor cells introduced in the opposite media channel may invade through the ECM and intravasate across the opposing endothelium. Tumor cells introduced in the endothelial channel may extravasate by transmigrating across the endothelium and invading the ECM. **(C)** Endothelial cells premixed with the ECM may self-organize into a vessel network of capillaries and microvessels. The microvessels may anastomose with the separate media compartments and permit flow. Tumor cells introduced into one compartment may be circulated through the microvessel network, adhere to the vessel walls, and extravasate.

As a variation of the predefined scaffold model, ECM is deposited between two microfluidic channels and a monolayer of endothelial cells formed on the exposed ECM in one channel, while tumor cells are seeded in the opposing channel (Figure 5). These devices have been used to image invasion, intravasation, extravasation, and tumor-driven angiogenesis under static flow conditions and have the potential to be used as high-throughput screens for cancer invasiveness (Zervantonakis et al., 2012; Jeon et al., 2013; Lee et al., 2014).

### Microvessel Self-Assembly

The aforementioned devices establish the vessel endothelium by seeding endothelial cells on predefined ECM surfaces and are thus limited to generating microvessels typically larger than 50 μm in diameter due to limitations in uniform cell seeding. To achieve microvessels on the capillary scale, approximately 10–20 μm in diameter, endothelial cells can be embedded within a matrix and allowed to self-assemble into a random vessel network (Figure 5). Perfusion through these vessels can be established once the networks have connected or anastomosed with nearby media ports or channels (Moya et al., 2013). Once perfusion is established, tumor cells may be circulated through the small diameter vessels, adhere to the vessel walls, and extravasate into the surrounding ECM (Chen et al., 2013). Tumor cells embedded within the surrounding ECM on the periphery of self-assembled vessel networks can be both activated or arrested in a dormant state due to interactions with the vessel endothelium (Ghajar et al., 2013).

## Summary and Future Prospects

*In vitro* models allow researchers to recapitulate aspects of the tumor microenvironment using specific cell types, extracellular matrices, and soluble factors. Controlling the various components of the model enables investigation of interactions within the tumor microenvironment, as well as the response to stimuli such as chemotherapeutics. There is a wide range of tumor models, each with distinct advantages and disadvantages. Due to the inherent differences in complexity and functionality, the choice of model is usually dependent on the application. A disadvantage of the wide range of tumor models is the lack of standard protocols and the difficulty in comparing results from different models. This problem will be exacerbated by the increase in the range and complexity of models available to researchers.

Transwell-based models are widely used to study migration and invasion of cancer cells across a porous membrane, or intravasation or extravasation across an endothelial monolayer, in a simple, high-throughput 2D platform. The next generation of transwell models will incorporate patient-specific cells to assay migration potential as a diagnostic tool. The incorporation of target cancer cells or biopsy samples in the basolateral chamber is a high-throughput approach to combining assessment of drug transport, uptake, and efficacy in a single assay.

Multicellular tumor spheroids recapitulate the 3D architecture and transport phenomena of tumor tissues and can be used to investigate growth and proliferation of tumor tissues, invasion into ECM, angiogenesis, immune interactions, and drug screening. Spheroids are able to recapitulate the basic 3D structure of tumors, including multicellular structure, central necrosis, and proliferation gradients depending on tumor type. The next-generation spheroid models will likely exploit advances in embedding in ECM and coculture with other cell types, such as immune cells, to elucidate immune cell interactions. Microfluidic devices are increasingly used to form spheroids and perform rapid drug screening.

Hybrid models, such as embedded *ex vivo* tumor sections, are useful for investigating tumor growth, invasion, matrix remodeling, and drug screening using patient biopsies. 3D invasion models, in which single cells are embedded in a 3D ECM, are used to study invasion, matrix remodeling, angiogenesis, and dormancy. Avascular microfluidic models are used to study the tumor microenvironment, including migration and extravasation. Hybrid models, in particular embedded tumor sections and 3D invasion models, are well suited for patient-specific drug screening and predicting outcomes.

Tumor-microvessel models build on the complexity of avascular microfluidic models by introducing a vessel component and are particularly well suited for modeling tumor–vessel interactions over time and investigating angiogenesis, vessel-induced tumor cell dormancy, intravasation, or extravasation. Current tumor-microvessel models represent a reductive approach to studying metastasis, where at a minimum, a functional vessel lined with endothelial cells and cocultured with tumor cells is required. Sustained perfusion of the vessels improves their physiological relevance and their adaptability to live cell imaging permits the study of the mechanistic details behind intravasation and extravasation. Recent advances in quantifying gene expression within these models may be applied to understanding the biochemical interactions between vessels and tumor cells (e.g., angiocrine and tumor paracrine signaling) that determine tumor cell proliferation and dormancy and govern tumor-driven angiogenesis. Future work may improve our understanding of the tumor microenvironment and cancer progression through the manipulation of physical cues, such as shear stress and interstitial flow, introduced through the vessel and the additional coculture of other relevant cell types within the surrounding matrix (e.g., tumor-associated macrophages, neutrophils, and fibroblasts).

In summary, advances in tumor cell biology, 3D cell culture, tissue engineering, biomaterials, microfabrication, and microfluidics have enabled rapid development of *in vitro* tumor models. Variations of traditional models are characterized by increased complexity through the incorporation of multiple cell types (coculture), ECM materials, and spatial and temporal introduction of soluble factors. Other innovations include incorporation of vessels to introduce tumor vasculature, since these leaky tumor vessels tend to influence cancer progression and drug transport. The development of 3D tumor culture systems is bridging the gap between *in vitro* and *in vivo* methods for drug screening as *in vitro* 3D models continue to develop to be better indicators of *in vivo* drug efficacy.

The drive toward precision medicine has resulted in increased interest in adapting *in vitro* tumor models for patient-specific therapies, clinical management, and assessment of metastatic potential. The next generation of *in vitro* tumor models will include combinations of existing models and the incorporation of new technologies for specific applications. In particular, developments in the field of organogenesis in combining a source of precursor cells that self-organize into a specific tissue or organ may be exploited in new tumor models.

## Author Contributions

MK, AP, AW, ZX, and PS reviewed and evaluated the literature and wrote the article.

## Conflict of Interest Statement

The authors declare that the research was conducted in the absence of any commercial or financial relationships that could be construed as a potential conflict of interest.
